# Supplementary material for: Phytogeographic Characteristics of Montane Coniferous Forests of the Central Balkan Peninsula (SE Europe)
Source: Plants (Basel). 2022 Nov 22;11(23):3194. doi: 10.3390/plants11233194 (PMC9741231; doi:10.3390/plants11233194)
Supplement: Supplementary file 1 [file plants-11-03194-s001.zip › Supplementary Materials Figures S1-S5-plants-2015086.pdf]

Supplementary Figures. Distribution of plots by decades

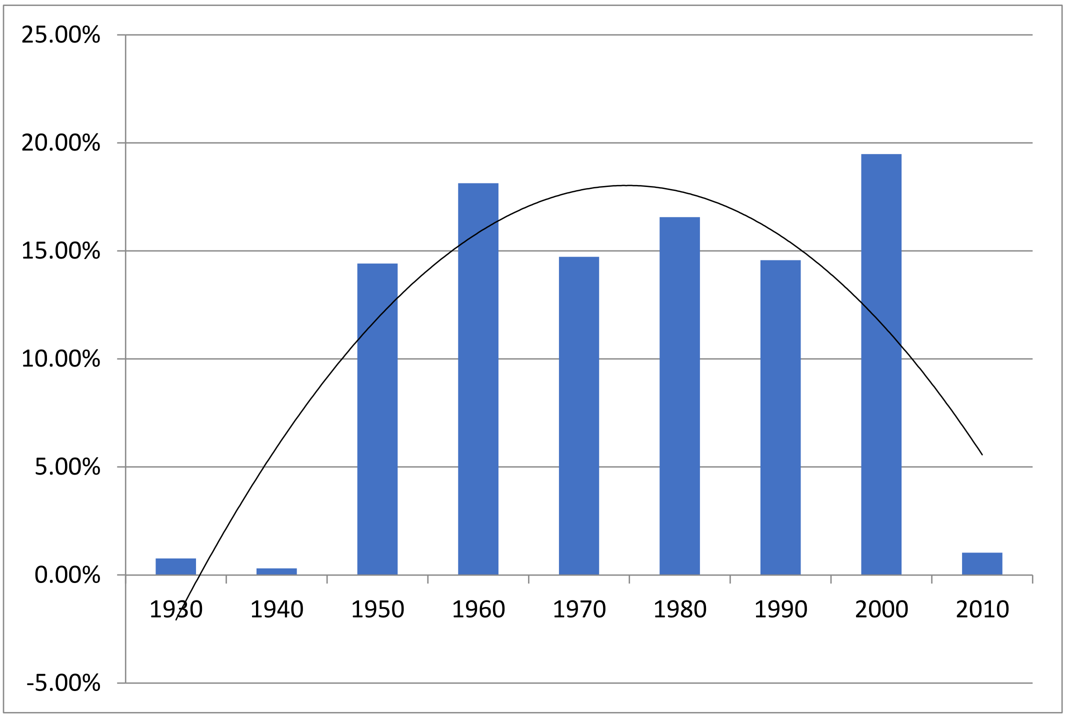

Figure S1. Distribution of plots by decades for all mountain systems

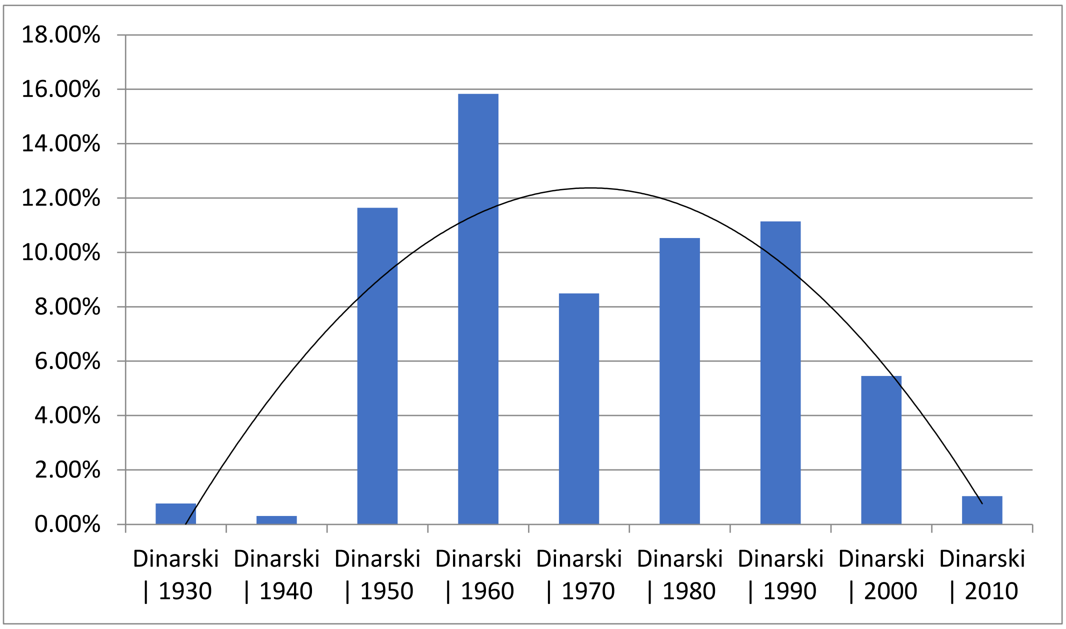

Figure S2. Distribution of plots by decades for Dinarides.

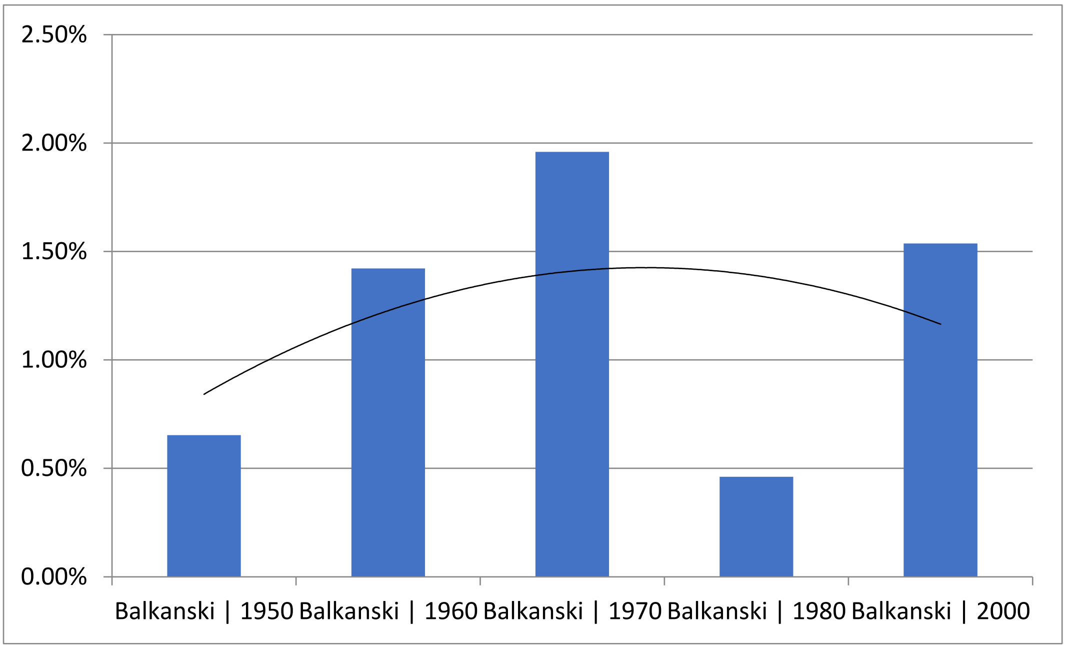

Figure S3. Distribution of plots by decades for Balkan (Stara planina) mountains.

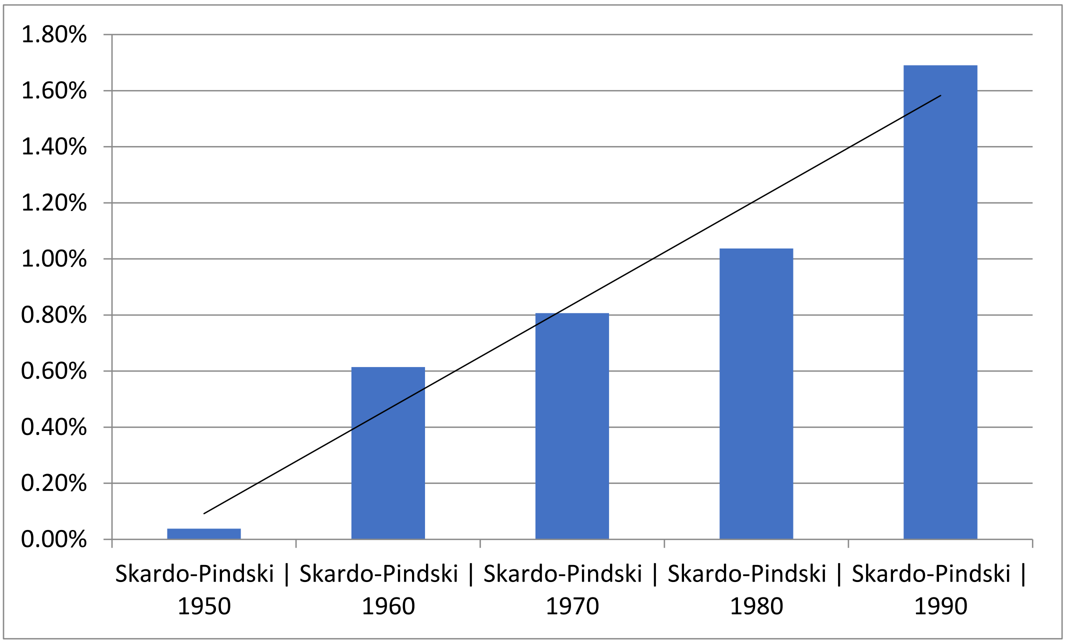

Figure S4. Distribution of plots by decades for Scardo-Pindic mountains.

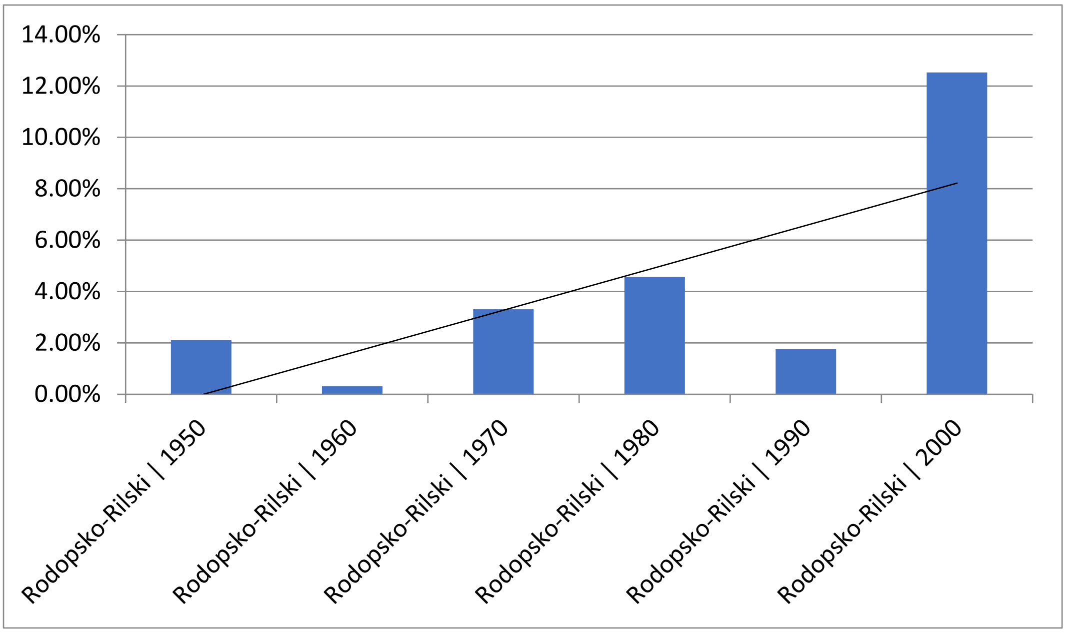

Figure S5. Distribution of plots by decades for Rhodope mountains.
